# Corporations and financial institutions driving coastal conflicts involving Indigenous Peoples

**DOI:** 10.1007/s13280-026-02406-x

**Published:** 2026-05-13

**Authors:** Sebastian Villasante, Julia Tovar Verba, Veronica Relano, Sílvia Gómez, José Bakit, Nathan J. Bennett, Anthony Charles, Rafael Calderón-Contreras, Andrés M. Cisneros-Montemayor, Bernardo M. Flores, Lucas A. Garibaldi, Maria Grazia Pennino, Priscila F. M. Lopes, Unai Pascual, Nick Roskruge, Adriana Carolina Flores-Díaz, Yesenia Hernández Márquez, Karen O’Brien

**Affiliations:** 1 EqualSea Lab-CRETUS, University of Santiago de Compostela, 15782 A Coruña, Spain; 2 Department of Social and Cultural Anthropology, Autonomous University of Barcelona, 08193 Bellaterra, (Cerdanyola del Vallès), Spain; 3 Departamento de Acuicultura, Facultad de Ciencias del Mar, Universidad Católica del Norte, Coquimbo, Chile; 4 Global Science, WWF, Washington, USA; 5 People and the Ocean Specialist Group, Commission On Environmental, Economic and Social Policy, International Union for the Conservation of Nature, Gland, Switzerland; 6 Institute for the Oceans and Fisheries, University of British Columbia, Vancouver, Canada; 7 Department of Environmental Science and Sobey School of Business, Saint Mary’s University, Halifax, Canada; 8 Departamento de Ciencias Sociales, Universidad Autónoma Metropolitana, Unidad Cuajimalpa, Mexico City, Mexico; 9 School of Resource and Environmental Management, Simon Fraser University, Burnaby, Canada; 10 Instituto Juruá, Manaus, Brazil; 11 Instituto de Investigaciones en Recursos Naturales, Agroecología y Desarrollo Rural, Universidad Nacional de Río Negro, Río Negro, Argentina; 12 Instituto de Investigaciones en Recursos Naturales, Agroecología y Desarrollo Rural, Consejo Nacional de Investigaciones Científicas y Técnicas, Río Negro, Argentina; 13 Institut de Ciències del Mar, Consejo Superior de Investigaciones Científicas (ICM-CSIC), P. Marítim de la Barceloneta, 37-49, 08003 Barcelona, Spain; 14 Department of Ecology, Fishing Ecology, Management and Economics Group, Universidade Federal do Rio Grande do Norte, Natal, 59078-900 Brazil; 15 Research Institute of the University of Bucharest (ICUB), 010041 Bucharest, Romania; 16 Institute of Biological Research Cluj, National Institute of Research and Development for Biological Sciences, Cluj-Napoca, Romania; 17 Basque Centre for Climate Change, Scientific Campus of the University of the Basque Country, 48940 Leioa, Spain; 18 Ikerbasque, Basque Foundation for Science, 48009 Bilbao, Spain; 19 Te Atiawa iwi, Tahuri Whenua National Maori Horticultural Collective, Manawatū, Aotearoa, New Zealand; 20 Transdisciplinary University Centre for Sustainability, Universidad Iberoamericana, Mexico City, Prol. Paseo de la Reforma 880, Lomas de Santa Fe, 01219 Álvaro Obregón, Mexico City, Mexico; 21 Rueda de Medicina A.C. ADE S.C., San Pedro Garza García, Mexico; 22 Climate Change Leadership Research Unit, Uppsala University, Uppsala, Sweden; 23 Department of Sociology and Human Geography, University of Oslo, Oslo, Norway; 24 The Anthropocene Laboratory, Royal Swedish Academy of Sciences, Lilla Frescativägen, 4A 114, 18 Stockholm, Sweden

**Keywords:** Coastal zones, Extractive and development activities, Finance, Indigenous Peoples, Socio-environmental conflicts

## Abstract

Socio-environmental conflicts associated with large-scale economic projects in Indigenous territories are well documented, but conflicts in coastal areas remain largely unexplored, despite the crucial differences between inland and coastal ecosystems. While the empirical literature about socio-environmental conflicts in Indigenous territories in coastal areas is growing, the role of financial institutions and corporations in these conflicts is often overlooked. This study addresses this gap by examining the financial institutions and corporations associated with more than 400 reported socio-environmental conflicts affecting Indigenous Peoples in coastal areas worldwide. Our results show that most financial institutions supporting projects linked to conflicts are based in West Europe and North America, with North American financial institutions linked to multiple projects. The energy sector accounts for the highest number of socio-environmental conflicts, particularly those involving corporations operating across continents. Many European and North American corporations operate mostly in Africa, but also globally. Corporations with overseas operations are associated with a higher intensity of conflicts. These findings highlight the need to scrutinize financial investments and corporate practices driving socio-environmental conflicts in coastal Indigenous territories, where human rights are often at risk. The lack of due diligence and accountability in applying environmental and social safeguards weakens protections for both ecosystems and Indigenous communities. Strengthening the enforcement of national and international laws on environmental sustainability, human rights and Indigenous rights is therefore essential for coastal development projects.

**Supplementary Information:**

The online version contains supplementary material available at https://doi.org/10.1007/s13280-026-02406-x.

## Introduction

In recent decades there has been a notable global increase in commercial interest in coastal areas and the ocean (Bennett et al. 2022b; Charles 2025). This interest stems not only from the traditional roles in providing food, transport and trade, but also from the recognition of these spaces as a source of strategic economic resources, such as minerals and fossil fuels (United Nations Conference on Trade and Development 2023). The new economic and extractive frontier is attracting significant global financial investments, driving large-scale coastal industrial developments (Jouffray et al. 2020), at the expense of traditional communities and users, including Indigenous Peoples and small-scale fisher communities. Historically, coastal areas have been the frontiers for colonization, and coastal Indigenous Peoples have often been the first to be impacted by such invasions (O’Fallon and Fehren-Schmitz 2011; Lightfoot et al. 2013). As a result, coastal Indigenous Peoples were also the first to suffer massive population collapse, slavery, as well as displacement and degradation of their ancestral lands and seas (O’Fallon and Fehren-Schmitz 2011). Generally oceans continue to be associated with weaker recognition as regards Indigenous Peoples’ rights, as compared with inland territories, likely due to economic pressures (O’Fallon and Fehren-Schmitz 2011; Capistrano and Charles 2012; Hoskins et al. 2025). These particularities, combined with the higher level of environmental vulnerability of coastal regions due to climate change (Cruz-Ramírez et al. 2024) and industrial overfishing (Pauly et al. 2005), make it necessary to better understand how the diverse external and internal economic, environmental and biocultural interests interact in coastal Indigenous territories.

Activities such as mining, oil and gas extraction, and large-scale infrastructure development projects rank among the most heavily financed investments worldwide. Whether on the ocean, coast or land, these economic activities are frequently located in or adjacent to Indigenous territories, where historical marginalization has often limited Indigenous Peoples’ own use of their territories, lands, and water and impacted their political power to decide on local development in their own terms (Garnett et al. 2018; Schuster et al. 2019; Sze et al. 2022; Kennedy et al. 2023; Reyes-García 2025; Villasante et al. 2025a). These projects typically receive backing from a diversity of financial institutions (FIs) such as private banks, multilateral development banks, and investment funds—including those that have publicly committed to Environmental, Social, and Governance (ESG) and sustainable finance principles of Performance Standards or Frameworks (International Finance Corporation 2012). Although these principles establish measures and requirements to prevent impacts on Indigenous Peoples (7), biodiversity (6) or lands (5) (see e.g. Performance Standards 6, 5 and 7 at the ESG framework) (International Finance Corporation 2012), they are often not respected. Between 2016 and 2021, for example, more than USD 3.8 trillion were invested in fossil fuel companies, many of which operated without securing proper consultation or consent from affected Indigenous communities in their territories (UNDP 2019; United Nations 2019; Rainforest Action Network 2022). Such financing practices contradict the principles of Free Prior and Informed Consent (FPIC) established in the United Nations Declaration on the Rights of Indigenous Peoples (UNDRIP 2007), undermining Indigenous land rights, self-determination, and ecological stewardship. Corporate and financial initiatives are often carried out without FPIC, and even other “due diligence measures” are not standardized or applied, and, when they are acknowledged, it is often through questionable methods (e.g. not providing complete information, without using the local language, or following shallow ‘check-box’ approaches) (Anaya 2008; UNHR 2023). Continued investments by financial and corporate actors in extractive activities and sectors in Indigenous coastal territories without proper regulation would perpetuate environmental degradation, erode human rights, and exacerbate existing and future socio-environmental conflicts (Ayling and Gunningham 2017).

The accelerating expansion of externally funded development projects and industrial activities into peripheral and rural regions worldwide has led to severe consequences for Indigenous Peoples and local communities, from displacement and loss of traditional livelihoods to cultural disruption and increased violence against ocean defenders (Bennett et al. 2023, 2022a; Scheidel et al. 2023; Llavero-Pasquina 2025). These impacts are often magnified in cases involving multinational corporations operating internationally, where they generate impacts on the health and well-being of Indigenous Peoples resulting in human rights violations (Bennett et al. 2023; Llavero-Pasquina 2025). In an intersectional context, Indigenous women in particular face compounded vulnerabilities due to gender-based discrimination and systemic exclusion from decision-making processes (ILO 2019; Tran and Hanaček 2023).

While large FIs are engaged in a myriad of development projects around the world, historically they have not considered the social and environmental impacts of their investments adequately. Some FIs state that they are taking steps to address these recurring issues, through ESG frameworks, human rights consideration, Indigenous Peoples Policy Frameworks, and efforts to align capital flows with sustainability and human rights goals (OECD 2020). In addition, the Paris Agreement indicates that financing should be directed toward lower greenhouse emissions and more resilience to climate change (UNFCCC 2015). The Global Biodiversity Framework (GBF), adopted in December 2022 under the Convention on Biological Diversity (CBD), calls for eliminating, phasing out, or reforming incentives, including subsidies, harmful to biodiversity, in a proportionate, just, fair, effective and equitable way, starting with the most harmful incentives (Target 18) (CBD 2022). Achieving these goals requires aligning funding schemes with rights-based conservation and ensuring that investments do not exacerbate biodiversity loss and social and cultural harm. However, without restructuring sustainable finance mechanisms to strengthen Indigenous leadership, enforce FPIC, and prioritize community-driven approaches, needed transformative potential risks being undermined (Xu et al. 2021; Chan et al. 2023).

Some FIs have been taking steps to address these problems over the last decade. For instance, the World Bank has developed the Indigenous Peoples Policy Framework and, more recently, the Indigenous Peoples’ Resilience Framework, which recognizes the vital role of Indigenous Peoples in conservation and aims to support their self-determination and climate resilience (World Bank 2024c). Nevertheless, FIs and corporations still need to align their portfolios with global commitments such as Kunming-Montreal GBF, the Paris Agreement, the UN Declaration on the Rights of Indigenous Peoples, the ILO Convention 1989 (nº 169) and the Universal Declaration on Human Rights (UNDRIP 2007; UNHR 2023). Therefore, adopting and implementing stringent due diligence mechanisms and safeguards is urgent to prevent financing and implementing projects that adversely affect Indigenous Peoples’ territories, ecosystems and rights (UNFCCC 2015; CBD 2022).

It is clear that financing development projects in coastal areas has primarily served the interests of investors in the Western world (Schutter et al. 2024), even when projects are framed as using sustainability approaches. Beyond failing to serve those most in need of support, however, these investments can also cause active harm to vulnerable populations such as Indigenous Peoples. Although violence against Indigenous Peoples, as one of the main dire expressions of social-environmental conflicts, is well documented (Scheidel et al. 2020), as are the broader impacts of large-scale development projects on Indigenous territories, environments and rights (Garnett et al. 2018; McGregor et al. 2020; Scheidel et al. 2020, 2023; Villasante et al. 2025b), the role of public and private FIs (e.g., banks, investment firms) and the corporations in socio-environmental conflicts remains largely unknown. Understanding the institutions financing these projects, the corporations conducting, and the geographical origins of these actors, is essential for ensuring accountability and aligning financial systems with environmental justice and the rights of Indigenous Peoples.

This study presents a global-scale picture of FIs and corporate actors involved in socio-environmental conflicts, which here refers to a threat resulting from different sectors or activities in the coastal environment, affecting Indigenous Peoples, and leading to a struggle and/or social mobilization. More specifically, we identify the financial and corporate actors linked to conflictive projects, identify the most affected Indigenous groups, visualize the geographic patterns of actors origins and projects’ locations, the intensity of the conflicts, and the economic sectors mostly represented in the conflicts. The projects were classified in ten sectors: energy production, mineral extraction, biomass and land, industrial, infrastructure, tourism, biodiversity and conservation, water management, waste management, and nuclear. Our results highlight how the growing number of such activities, together with the increasing level of industrial concentration and investment, is posing mounting risks to coastal environments and the territories and rights of Indigenous Peoples.

## Results

### Characterization of conflicts: Geographical location, sectors and affected ethnic groups

**Fig. 1:**
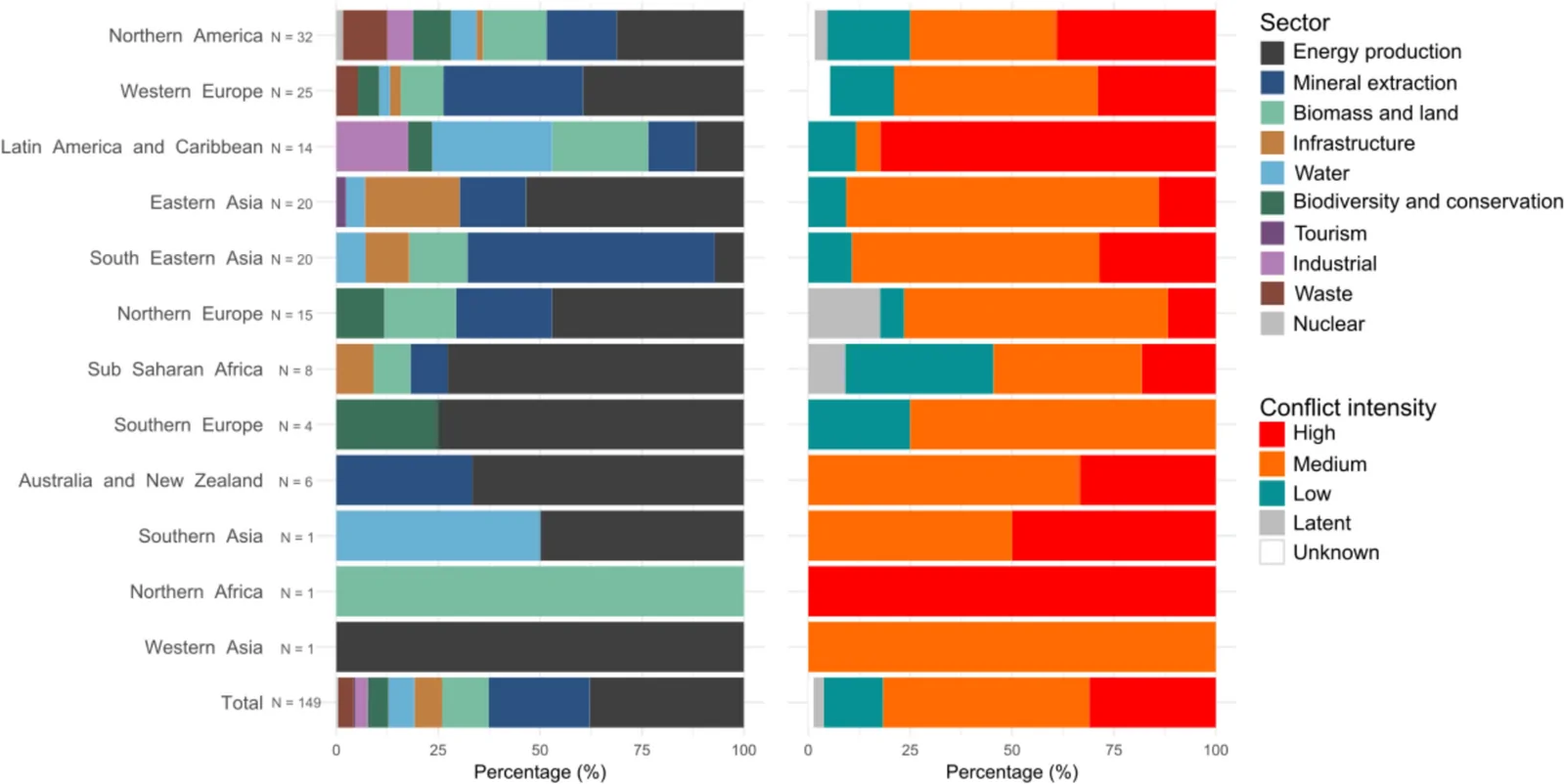
**a** Percentage of conflicts by sector (energy production, mineral extraction, biomass and land, infrastructure, water management, biodiversity and conservation, tourism, industrial, waste management and nuclear) and **b** level of intensity (high, medium, low, latent or unknown) per origin of the FIs (UN Nations regions). *N: total number of cited FIs per region

To identify the financial and corporate actors associated with socio-environmental conflicts affecting Indigenous Peoples in coastal areas, we first built a dataset based on the Environmental Justice Atlas (EJAtlas, see Supplementary Material for a detailed explanation of the methodology). We followed the concept used in the EJAtlas for socio-environmental conflict, that consists in struggles or mobilizations where civil society challenges threatening projects or activities causing perceived social and environmental harm (Temper et al. 2015, 2018). These conflicts are often driven or made worse by environmental injustices and structural inequalities involving struggles over power, participation, benefits distribution, and recognition of diverse worldviews (Temper et al. 2015, 2018). Each conflict case is a documented concrete situation where the civil society makes explicit claims against a specific project, policy or economic activity due to its social and environmental damages (Temper et al. 2015, 2018). Our selected dataset consists of 401 cases in 72 countries, associated to at least 275 ethnic groups (Supplementary Material Table S1). South East Asia and Latin America and the Caribbean were the most cited regions with occurrence of the conflicts, with a combined total of 189 conflicts. Conflicts were classified in 10 different sectors, with Energy production (*n* = 126), Mineral extraction (*n* = 74) and Biomass and land conflicts (*n* = 72) being the most cited ones (see Fig. 1 for the complete list). Among the socioeconomic impacts caused by the conflicts, the most documented were loss of livelihood, human rights violations, and loss of sense of place. In terms of intensity, the conflicts were mostly characterized as medium (*n* = 164; 40%) or high (*n* = 127; 31%).

### Financial institutions

#### Geographic origin and sectors

To identify the FIs supporting coastal projects linked to conflicts impacting Indigenous Peoples in coastal areas, and the geographic distribution of origin and impacted groups, we analyzed the FIs cited in the dataset. We identified 149 FIs providing direct or indirect support to development projects affecting Indigenous Peoples and generating 85 coastal socio-environmental conflicts, from a larger set of 401 cases recorded (Supplementary Material Table S2). For the remaining 316 conflicts in the dataset, no information on FIs was reported. The identified 149 FIs were primarily located in North America (32 institutions cited in 42 cases in total) and Western Europe (25 institutions cited in 26 cases), and mostly related to conflicts associated with energy production, mineral extraction and biomass and other related land conflicts of high or medium intensity (Fig. 1, Supplementary Material Table S2).

The 20 FIs from East Asia, cited in 24 cases, are mostly related to the energy production sector, and, at a lower frequency, with infrastructure projects. Financial institutions from South East Asia, cited in 14 cases, are mostly supporting projects of mineral extraction. The 15 FIs from North Europe are mostly linked to the energy production sector, and also with mineral extraction projects. The 14 FIs based in Latin America and the Caribbean, cited in 11 cases, were linked mostly to projects around water management, biomass and land, and industrial and utilities conflicts (Fig. 1).

Financial institutions based in Latin America and the Caribbean, in Southern Asia, and Northern Africa are the ones associated with a larger percentage of high-intensity conflicts, while projects supported by FIs from North America, Sub-Saharan Africa and South Europe showed a higher share of low-intensity conflicts, in comparison with the other regions (Fig. 1). The five most frequently mentioned FIs supporting activities linked directly or indirectly to coastal socio-environmental conflicts affecting Indigenous Peoples were the World Bank (*N* = 16), the International Finance Corporation (IFC), a member of the World Bank Group (*N* = 5), the Inter-American Development Bank (IDB, *N* = 5), the European Investment Bank (EIB, *N* = 4), and the European Commission (*N* = 4). Of the 39 FIs associated with multiple conflicts, nine are based in North America (cited in 32 conflicts in 23 countries), and nine are based in East Asia (cited in 20 conflicts in 14 countries). Most FIs (*N* = 110) were cited in only one conflict each, while 39 FIs were associated with multiple projects.

#### Affected ethnic groups

**Fig. 2:**
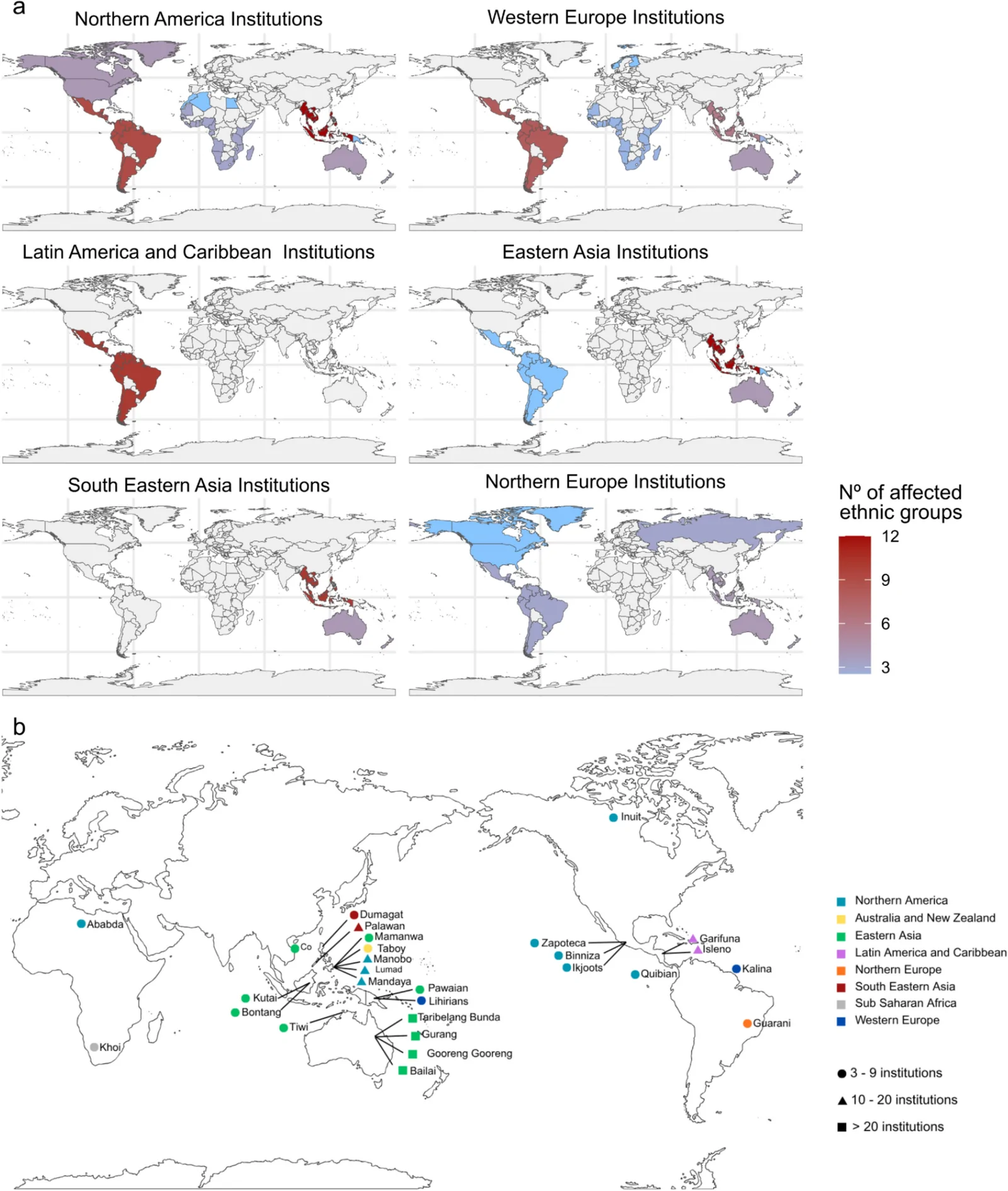
**a** Number of ethnic groups directly affected by socio-environmental conflicts linked to FIs from each UN region (the six regions with higher numbers of impacted groups); **b** Geographic location and names of the ethnic groups affected by conflicts linked to three or more (for better visualization) FIs, colored by region of origin (eight regions with more FIs). The amount of FIs associated with projects related to conflicts affecting each group is represented by symbols (circle—3 to 9 FIs, triangle—10 to 20 institutions, square—more than 20 institutions). The colors indicate the origin of the FIs (See Table S2 in the Supplementary Material for all ethnic groups directly affected by socio-environmental conflicts)

To investigate the ethnic groups associated with the socio-environmental conflicts, we first listed the number of affected groups linked to each financial institution, and identified the specific groups involved. Conflicts associated with FIs based in North America and Western and North Europe account for the highest number of affected ethnic groups, spanning a wide range of countries, predominantly in Latin America and the Caribbean, Sub-Saharan Africa, South East Asia and Oceania (Fig. 2a, Supplementary Material Table S3). In contrast, conflicts linked to FIs based in East Asia mainly impact ethnic groups in Asia, Oceania, and, at a lower level, Latin America and the Caribbean. Those conflicts linked to FIs with headquarters in South East Asia and Latin America and the Caribbean tend to primarily affect ethnic groups within their own regions (Fig. 2a).

The ethnic groups affected by socio-environmental conflicts involving a larger number of FIs are located in Australia and New Zealand region and include the Bailai, Gooreng Gooreng, Gurang, and Taribelang Bunda Indigenous Peoples (Fig. 2b, Supplementary Material Table S3). The ethnic groups affected by socio-environmental conflicts involving between 10 and 20 FIs include the Garifuna and Isleño peoples in Latin America and the Caribbean, as well as the Palawan, Manobo, and Lumad peoples in Asia (Fig. 2b). South Eastern Asia is the region with the highest number of ethnic groups impacted by conflicts linked to multiple FIs (Fig. 2b). A large number of the listed ethnic groups are affected by projects supported by the World Bank. These include cases affecting the Dumagat in the Philippines, the Garifuna and Isleno in Nicaragua and Honduras, the Zapotec in Mexico, the Guarani in Brazil, the Lumad in the Philippines, and several groups in Australia.

### Corporations

#### Intercontinental dynamics of operations

**Fig. 3:**
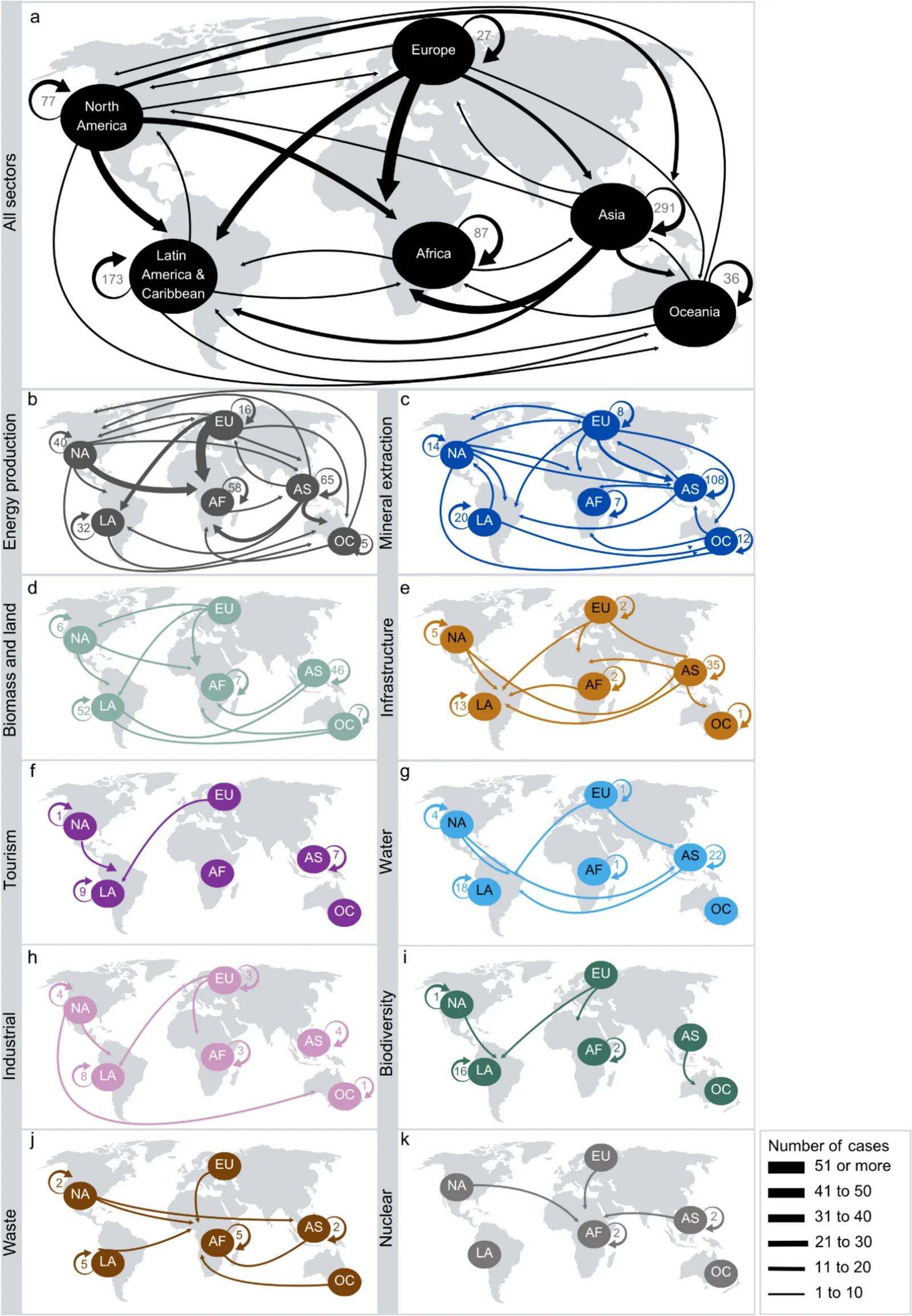
Geographical dynamics of corporate headquarters and projects operations locations. **a** Number of associated conflicts in all economic sectors impacting Indigenous Peoples in coastal areas, and for specific sectors, including, **b** Energy production, **c** Mineral extraction (mineral ores and building materials extraction), **d** Biomass and land conflicts (forests, agriculture, fisheries and livestock management), **e** Infrastructure and built environment, **f** Tourism recreation, **g** Water management, **h** Industrial and utilities conflicts, **i** Biodiversity and conservation conflicts, **j** Waste management and **k** Nuclear. Arrow thickness represents the number of conflicts. *Note NA—North America, LA—Latin America, EU—Europe, AF—Africa, AS—Asia, OC—Oceania

To investigate the geographical dynamic between corporate headquarters and operations, we listed the corporations cited in the dataset, identified their origins and linked them to the region of operation. There were 869 corporations from 81 countries identified as operating development projects involved in the 401 conflicts affecting Indigenous Peoples in coastal areas (Supplementary Material Table S4). In contrast with the findings on FIs, we found that the majority of the corporations’ origins (72%, *n* = 627) as well as the conflicts’ locations (78%, *n* = 311) are Latin America, Africa and Asia. Notably, Asia exhibited the highest number of cases involving Asian based firms (*n* = 100 from 115, Fig. 3a). Similarly, most projects (68%, *n* = 46) located in Europe or Northern America (total *n* = 67) were operated by corporations from those regions (Fig. 3a).

The larger number of cases of corporations operating in other regions are from European and North American firms operating in Africa (Fig. 3a). Latin America and Africa were the regions with more projects operated by corporations from other regions, with almost no cases of corporations from these regions operating in other regions (Fig. 3a, the complete list of corporations is in Supplementary Material Table S4).

Energy production generated the highest number of conflicts linked to projects involving corporations operating transcontinentally (*N* = 167, 42%, Fig. 3b). Mineral extraction also displayed strong transcontinental activity, particularly North American firms in Latin America, although the majority of cases worldwide still involved regional corporations (*N* = 169, 77%, Fig. 3c). In contrast, conflicts linked to biomass and land use were mainly driven by local corporations, though European corporations were notably involved in Africa (Fig. 3d). Latin America and Asia show the highest number of local biomass and land conflicts, with 52 and 46 cases, respectively (Fig. 3d).

A large proportion of infrastructure-related conflicts were operated by regional corporations (*n* = 58, 72%), but Latin America reported the largest number of foreign-operated projects, involving firms from North America, Europe, Africa and Asia (*n* = 10, 43%, Fig. 3d). Asia exhibited, again, a substantial number of local corporations operating projects linked to infrastructure-related conflicts, with 35 cases documented (87%, Fig. 3d). Tourism-related conflicts were less common and mostly concentrated in Latin America, involving both local (*N* = 9, 47%) and foreign (*N* = 10, 53%) corporations (Fig. 3e). Water-related conflicts were also concentrated in Latin America and Asia, largely local in origin (83%), with only a few involving corporations from other regions (Fig. 3f).

### Regional differences

**Fig. 4:**
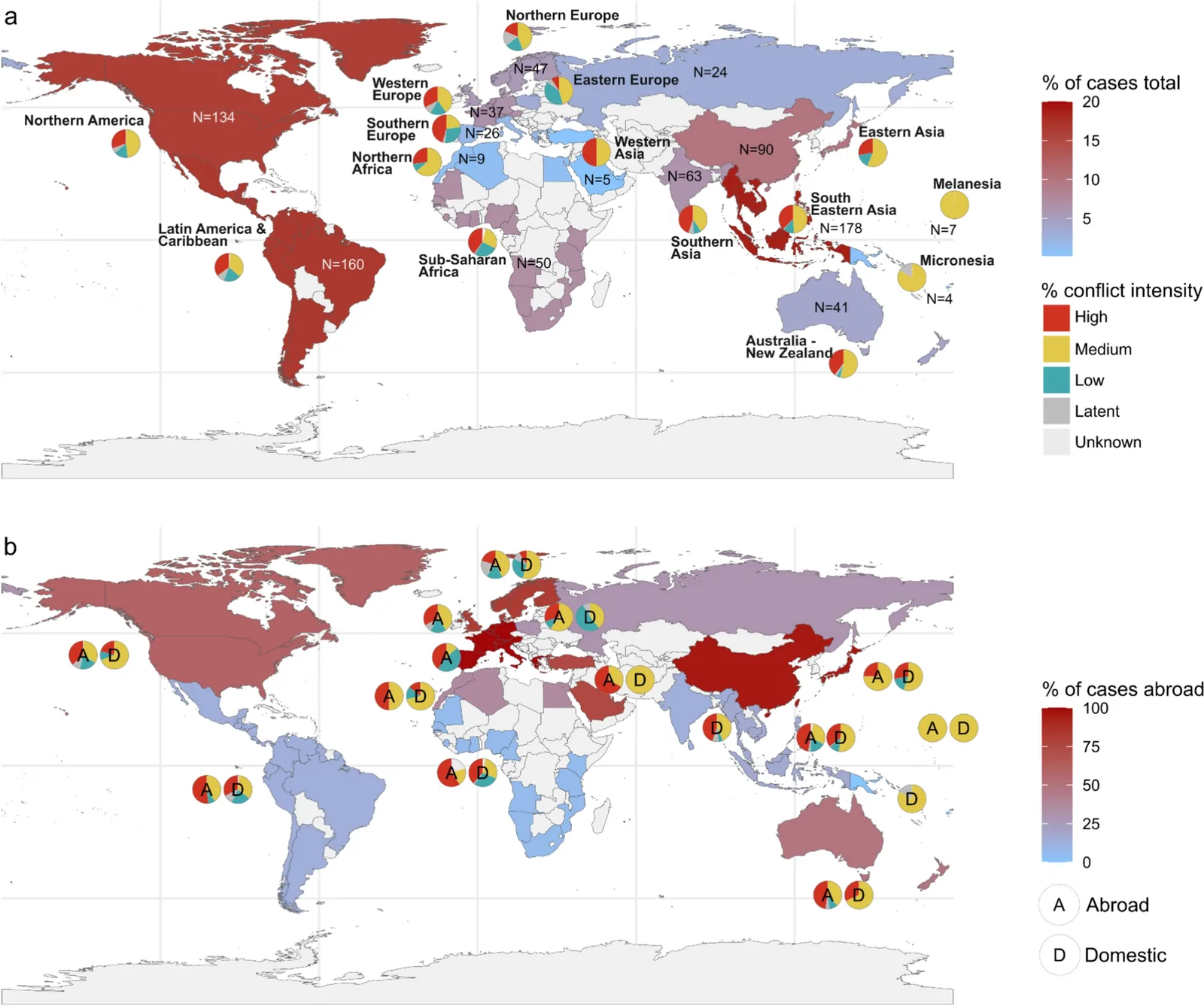
The regions of origin of corporations, following the geographic regions from the UN classification. **a** From the total number of cases, percentage of the ones associated with corporations and conflict intensity per region. Numbers (N) are the total number of corporations cited for each region. **b** Percentage of cases of corporations operating abroad relative to the total per region and conflict intensity for A—abroad cases or D—domestic cases

Some regions tended to dominate as the origins of corporations operating projects linked to conflicts (Fig. 4). South East Asia was the most frequent region of origin (199 corporations cited in 177 cases, 16%), followed by Northern America (133 corporations cited in 171 cases, 16%) and Latin America and the Caribbean (177 corporations cited in 159 cases, 15%, Fig. 4a). Corporations involved in conflicts abroad tended to be located in East Asia, Western Europe, North Europe and North America, while corporations from Latin America and the Caribbean, South East Asia and Sub-Saharan Africa were linked to domestic conflicts rather than those abroad (Fig. 4b). All regions followed the same pattern of conflict intensity: where corporations were operating abroad, the intensity of the linked conflicts tended to be higher than when the corporations were operating domestically (Fig. 4b).

**Fig. 5:**
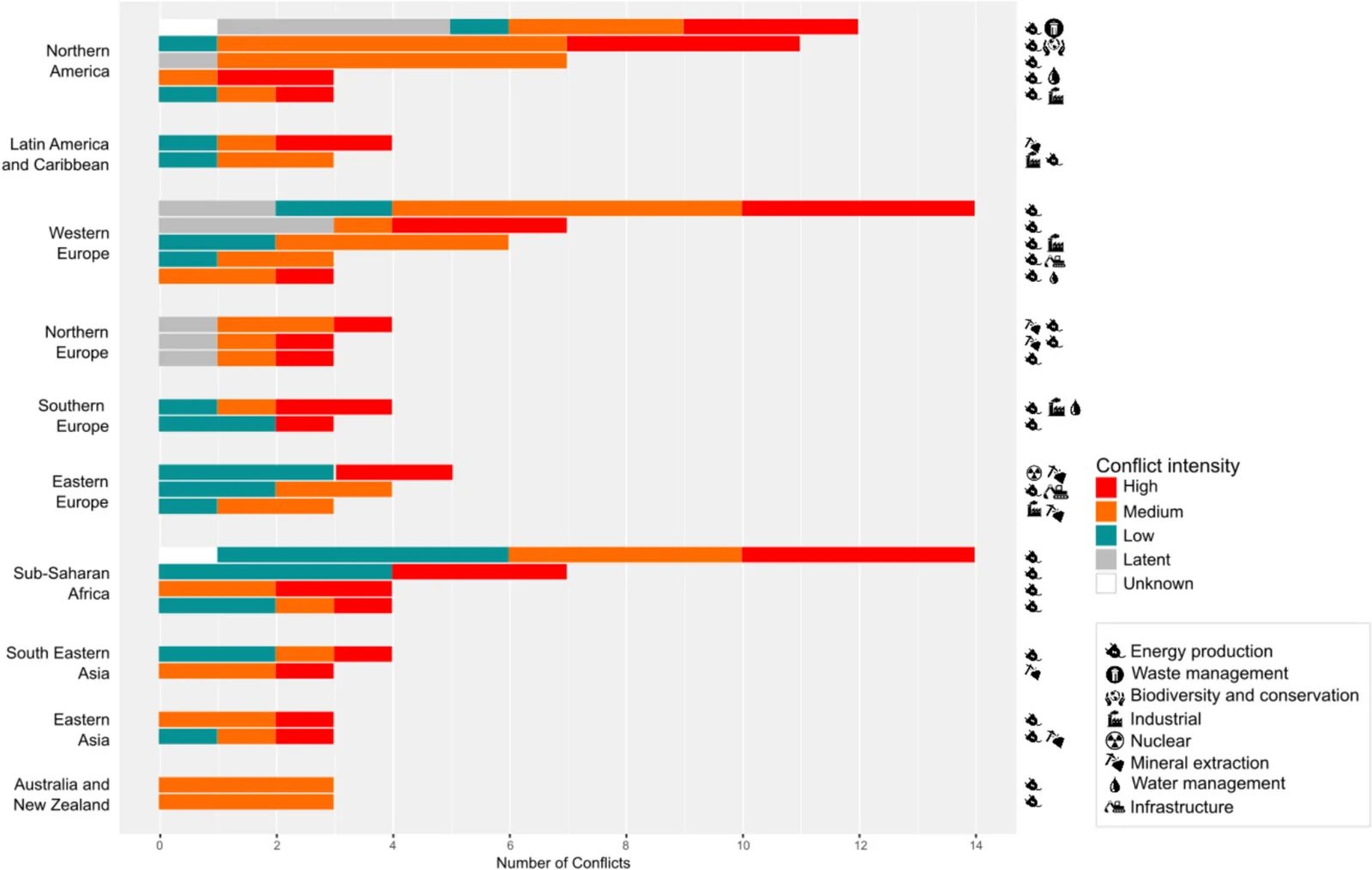
Number of socio-environmental conflicts linked to different corporations, distributed by origin of the corporation. Each bar represents one corporation, colors represent the conflict intensity—gray is latent, green low, orange medium, red high, white unknown—and symbols indicate the sectors to which the conflicts are related to

Two corporations involved in the highest number of projects linked to socio-environmental conflicts, both from the Energy production sector, are based in Western Europe and in Sub-Saharan Africa, each associated with 14 conflicts (Fig. 5). For the corporations from Western Europe, 10 out of 14 conflicts were of high or medium intensity, while for the corporations from Sub-Saharan Africa, 8 out 14 were of high or medium intensity. The regions with the highest number of corporations involved in three or more conflicts, with at least one related to energy production, are Northern America, linked to a total of 36 conflicts, mostly of medium intensity, and Western Europe, linked to 33 conflicts (Fig. 5). These are followed by Sub-Saharan Africa, with four corporations involved in 29 conflicts, exclusively from the energy production sector, and Nort Europe and East Europe, with three corporations engaged across a wide range of sectors (energy production, mineral extraction, nuclear, infrastructure and industrial, Fig. 5).

When all sectors were included, no significant overall association was found between conflict intensity and whether the conflict involved domestic vs. foreign corporations (X-squared = 6.36, df = 4; *p* = 0.17). However, when disaggregating the data by sector, distinct patterns emerged regarding the level of conflict associated with domestic versus foreign corporations. In mineral extraction projects, high-intensity conflicts were associated with the presence of foreign corporations (X-squared = 8.80, df = 3; *p* = 0.03), meaning that conflicts involving foreign countries were more likely to be of high intensity. In biomass and other land-related conflicts, low-intensity conflicts were associated with foreign corporations (X-squared = 11.74, df = 4; *p* = 0.01), indicating that conflicts involving foreign corporations were more likely to be of low intensity. When restricting the analysis to the most frequently reported corporations engaged in energy production projects, low-intensity conflicts were associated with the presence of domestic corporations (X-squared = 13.23, df = 4; *p* = 0.01).

We found no statistically significant correlation between the conflict intensity and the number of corporations involved for the whole dataset (*p* = 0.14), nor when splitting by UN region (North Africa *p* = 0.29, Sub-Saharan Africa Kruskal–Wallis chi-squared = 4.925, df = 4, *p* = 0.29, North Europe *p* = 0.37, East Europe *p* = 0.93, South East Asia *p* = 0.46, Southern Asia *p* = 0.98, Melanesia *p* = 0.38, Australia and New Zealand *p* = 0.65, North America *p* = 0.39, Latin America and the Caribbean *p* = 0.70), or by the main sectors (Energy production *p* = 0.13, Mineral extraction *p* = 0.37, Biomass and land *p* = 0.86, Infrastructure *p* = 0.83). It was not possible to test the association for Eastern Asia, Micronesia, Polynesia and West Asia due to the low number of occurrences.

## Discussion

Indigenous Peoples in coastal zones have long been stewards of some of the world’s most ecologically significant and vulnerable marine and coastal ecosystems, including mangroves and coral reefs, among others. Through traditional knowledge systems and locally adapted management practices, they have sustained their lands, water and territories for generations (Garnett et al. 2018; Bennett et al. 2022a). These communities have also been threatened by colonialism for centuries, and the growing pressures from climate change, pollution, and extractive industries, exacerbating the vulnerability of Indigenous Peoples, creating urgent demands for equitable responses (IPBES 2019, 2024). Yet, in the absence of fair and inclusive financial mechanisms and adequate due diligence in the expansion of the coastal and ocean economy, Indigenous Peoples will continue to remain systematically marginalized and negatively impacted (Araos et al. 2020; Lyons et al. 2023).

This study reveals that coastal socio-environmental conflicts affecting Indigenous Peoples are disproportionately driven by FIs and corporations based in Europe and North America, often associated with multiple conflicts and widespread impacts across ethnic groups. In contrast, South East Asia emerges as a global hotspot, with the highest concentration of affected Indigenous communities, reflecting dense networks of regionally and internationally financed development pressures. The data show that international FIs, including multilateral development banks, emerge as key financiers of projects associated with coastal socio-environmental conflicts affecting Indigenous Peoples, despite their public commitments to environmental and social governance principles such as their International Financial Corporation (IFC) Performance Standards and Frameworks on Environmental and Social Sustainability (International Finance Corporation 2012). To retain legitimacy and contribute to global sustainability goals, these financial institutions should further align their portfolios with the Kunming-Montreal GBF, the Paris Agreement, the UN Declaration on the Rights of Indigenous Peoples and the ILO Convention 1989 (nº 169) and the Universal Declaration on Human Rights. While such standards may reduce some risks, the literature shows that these governance mechanisms can operate symbolically, offering legitimacy without ensuring meaningful accountability or structural change needed to avoid or mitigate socio-environmental conflicts (Goldman 2005; Banerjee 2008; Birss and Sirén Gualinga 2023). Moreover injustices may not only be a product of policy implementation gaps, but actually a result from frameworks that prioritize (shallow) procedural compliance over redistribution, biocultural rights recognition, or financial autonomy. The understanding of this tension emphasizes the need for binding rules, independent oversight, and Indigenous-led projects beyond voluntary safeguards.

### Financial institutions

Finance has the potential to be a key strategy to address global environmental and social sustainability challenges. Public and private FIs have an increasingly crucial role in economic development through capital mobilization into strategic sectors. However, these investment decisions can overlook essential considerations related to poverty alleviation, social equity and biodiversity. The World Bank’s first formal framework to safeguard Indigenous Peoples was introduced in 1982, yet evaluations show that these and other guidelines or performance standards or frameworks, specifically standard number 7, related to Indigenous Peoples have not been consistently applied (World Bank 1982; Eastwood 2011). Our study indicates that FIs are falling short in many places in supporting sustainable and equitable development. For instance, in our dataset, the World Bank appears frequently (10% of the total) in connection with coastal projects affecting Indigenous Peoples through biomass and land management, especially large-scale monoculture plantations leading to land expropriation. It is also linked to energy projects in coastal and marine regions, including fossil fuels and, to a lesser extent, wind farms often supported by the World Bank through the Global Environment Facility Trust Fund (GEF).

However, recently evolving frameworks of the World Bank and the EIB demonstrate a shift toward more responsible investments, which could largely reduce conflicts and harm to Indigenous Peoples in the future. For example, the inclusion of terms like equity and justice in the World Bank Group Gender Strategy 2024–2030 (World Bank 2024d) indicates a starting point for aligning the institution’s policies with broader commitments to fairness and social responsibility. While gaps between policy and practice exist, integrating human rights, equity and justice in their frameworks is an essential step forward for investments that achieve sustainability of ecosystems and justice for people (Bennett et al. 2022b). As discussed above on past ESG principles, however, these efforts must be supported by clear evidence of changes in practices that align with FPIC and devolve decision-making power to Indigenous communities. This study includes past conflicts, often predating current social frameworks, whose impacts persist over time, meaning recent policy shifts may not yet be reflected. While FIs may be adapting, the data suggest that growth-driven coastal investments continue to reproduce neocolonial dynamics, particularly through large-scale projects imposed with limited local participation (Eastwood 2011; Dhar et al. 2020; Contreras et al. 2023).

The IDB, created in the 1960s to accelerate development in Latin America and the Caribbean, is headquartered in the USA, its largest shareholder. In our analysis, we identified at least five IDB-financed coastal projects associated with socio-environmental conflicts affecting Indigenous Peoples worldwide, related to aquaculture projects, oil palm plantations, and renewable energy. Despite this, the IDB has started playing a role in financing climate mitigation, clean energy, and nature-based solutions, and has formally committed to the Paris Agreement. Since October 2021, it claims it has expanded opportunities for Indigenous organizations to access self-determined funding, a strategy endorsed internationally. To guide these engagements, the IDB’s operations are governed by its active Environmental and Social Policy Framework (ESPF). This framework includes the bank’s latest standards, with a specific Environmental and Social Performance Standard (ESPS 7) for Indigenous Peoples (IDB 2020). Although these actions show a positive intention from the IDB, the disparity between the investments in Indigenous-led projects and large corporations’ projects continues to be large, so the requirement for clear frameworks protecting Indigenous Peoples and local communities in the implementation of any activity should be reinforced.

The EIB has established formal frameworks to protect Indigenous communities, guided by its Environmental and Social Sustainability Framework (European Investment Bank 2022) and reinforced by its “Approach to Human Rights” (European Investment Bank 2023). Yet, our analysis reveals at least five projects supported by the EIB related to coastal conflicts that displaced Indigenous groups. For example, while the EIB promotes sustainability and FPIC, it faced non-compliance reports from hydropower projects impacting Indigenous communities in Nepal (IWGIA 2021). While FIs like the World Bank, EIB, and the IDB are primary sources of financial flows associated with potentially positive projects for coastal and Indigenous communities (Humphrey and Brugger 2020), given the sheer volume of their investments, as we have shown, some projects can result in negative outcomes or local conflicts (World Bank 2021). Hence, future projects must dedicate larger efforts to avoid causing harm to Indigenous Peoples in coastal zones and inland.

### Geographic flows of socio-environmental conflicts

Rapid economic expansion has historically driven biodiversity loss and the erosion of Indigenous and traditional communities’ well-being. Since World War II, the growth of international trade and multinational corporations has intensified extractive activities, often relocating impacts to Africa, Latin America, and Asia while retaining benefits in the Global North and, more recently, China (Ortiz-Ospina 2017; Llavero-Pasquina 2025). This pattern of ecologically unequal exchange creates “sacrifice zones” and raises critical environmental justice concerns (Martinez-Alier 2023).

Within our dataset of coastal projects resulting in conflicts affecting Indigenous Peoples, this intercontinental operational flow is particularly pronounced in activities related to energy production. Africa, in particular, hosts the highest number of projects developed by foreign corporations (Fig. 3a). Limited infrastructure in many African countries reduces the competitiveness of domestic corporations, reinforcing dependence on foreign capital (Harrison et al. 2014). However, several governments are taking steps to counter this trend. Zimbabwe, for instance, has announced plans to ban all lithium exports until 2027 (Reuters 2025b), Guinea is tightening regulations on foreign mining operations to stimulate domestic investment (Mining Technology 2024), while Ghana has officially transferred ownership of three major bauxite concessions to its national mining corporation concluding a months-long process aimed at strengthening state control over strategic mineral reserves (Reuters 2025a). In contrast to European, North American or Chinese firms, corporations from Latin America, Asia and Africa are predominantly engaged in projects within their own regions.

### Corporations and origins

Large corporations have long contributed to environmental degradation and human rights violations, particularly through fossil fuel extraction, mining, and large-scale infrastructure (Downey et al. 2010; Prechel and Zheng 2012; Saes et al. 2021; Llavero-Pasquina 2025). They often operate in low-income though resource-rich countries with weaker regulations, while being headquartered in high-income nations where the social-ecological impacts are more limited (Cole et al. 2017; Llavero-Pasquina 2025). Even where social and environmental regulations may be stronger, the strong influence exerted by corporations can enable flexibility and downgrade safeguards. This helps explain why North America hosts many corporations involved in both domestic and international conflicts, while South East Asia shows more domestically rooted conflicts linked to weaker enforcement capacity (Yustitianingtyas et al. 2025).

Two corporations stand out for being implicated in 14 conflicts each, both linked to energy production. One, based in Sub-Saharan Africa (Nigeria), operates primarily in domestic oil extraction. The other, based in Western Europe (Netherlands), is primarily associated with projects abroad, most notably in Nigeria. The Niger River Delta, long affected by oil spills, has suffered severe ecological degradation and loss of livelihoods, leading to its designation as a “maritime zone of sacrifice” (Oyedele and Stoddart 2025). The next three most frequently cited corporations in our dataset are all from Northern America (US), mostly operating abroad, and like those above, also focusing on oil production (Obi 2008).

Sector-specific analyses revealed contrasting patterns, reinforcing the need for analysis focused on individual sectors. In mineral extraction, high-intensity conflicts, often characterized by widespread large-scale mobilizations, were associated with projects operated by foreign corporations. A similar pattern emerged in activities linked with energy production. Notably, for corporations involved in multiple projects, low-intensity cases were more often associated with domestic operators. In contrast, biomass and land-use conflicts showed the opposite pattern, with low-intensity conflicts associated with foreign corporations. However, the latter may be explained by the fact that these conflicts tend to attract less international investment, and accordingly, they are dominated by domestic firms.

### Outlook and future pathways

Our study has shown that coastal conflicts involving Indigenous Peoples are often rooted in unequal access to resources and decision-making due to persistent unjust global asymmetries. These in turn can be traced back to a concentration of capital, corporate power, and financial decision-making in wealthier countries, with great implications not only for Indigenous rights but also coastal and marine biodiversity. Injustice was presented in different forms in our dataset: as unequal participation in decision-making processes (e.g., lack of FPIC), as unequal allocation of environmental harms and access to territories, lands and water, as the multiple cases of forced displacement, as failures in recognizing Indigenous Peoples’ biocultural rights, knowledge, or governance structures. Further research could focus on identifying the impacts of different types of injustice.

Here we highlight the need for systemic changes to address inequities through greater transparency and accountability in both the national regulations and the global economy. This includes mandatory disclosure requirements for FIs and donors, and binding safeguards to prevent harm in Indigenous territories. At the same time, divestment and regulatory policies should phase out harmful financing, while prioritizing Indigenous-led blue finance and conservation mechanisms. These actions would support the implementation of Targets 22 and 18 of the Kunming-Montreal GBF (CBD 2022). At the national level, this would require integrating FPIC into legal frameworks and reforming subsidies to redirect funding toward Indigenous-led coastal initiatives.

More recently, FIs align themselves with global agendas on biodiversity conservation, climate change mitigation and poverty alleviation. The World Bank has played a significant role catalyzing investments—particularly in renewable energy infrastructure and climate resilience—by developing instruments like green bonds (World Bank 2015), co-supporting multi-billion dollar programs like the Climate Investment Funds (World Bank 2024a), and administering multi-donor trust funds such as PROBLUE (World Bank 2024b) or PROGREEN (World Bank 2023), among others. However, as of 2025, under the current US administration, it has scaled back funding for climate change mitigation and energy transition projects (Sabin Center for Climate Change Law 2025).

The establishment of performance standards by FIs, corporations and governments represents a significant commitment to safeguarding Indigenous Peoples. The ongoing socio-environmental challenges highlight the next crucial step: enhancing the on-the-ground application and oversight of these important policies. International legal frameworks increasingly emphasize direct financial support to Indigenous Peoples. The United Nations Declaration on the Rights of Indigenous Peoples (UNDRIP 2007) affirms their rights to manage and benefit from their territories, lands and water. The CBD, through Article 8(j) and related provisions to 2030 work program and the Kunming-Montreal GBF, explicitly calls for direct funding to Indigenous Peoples with Target 22 requiring Parties to ensure equitable participation, access to justice, and adequate financial resources (CBD 2022). In our dataset, Indigenous Peoples are primarily identified as groups negatively impacted by externally financed development projects, without distinguishing whether they act as governing authorities or affected rights-holders. Future research could examine how territorial governance relates to financial flows, accountability, and biodiversity outcomes. Furthermore, despite managing vast land and marine areas, Indigenous Peoples receive a negligible share of conservation finance, only 0.74% of climate-related funding, with limited direct access (Rainforest Foundation Norway 2021). In coastal zones, funding often bypasses Indigenous governance, weakening culturally grounded solutions (Rights and Resources Initiative 2015). We posit that redirecting investments toward Indigenous-led initiatives and recognizing their authority in conservation frameworks would strengthen biodiversity protection, climate action, and equity.

Transforming financing systems in coastal Indigenous territories by redirecting investments toward Indigenous-led conservation and regenerative economies, while promoting the application of FIs’ standards to prevent these conflicts, could offer a pathway toward upholding rights, enhance ecosystem resilience, and support global climate and biodiversity goals. This would require moving beyond extractive models toward economies embedded in ecological cycles and long-term restoration (Čegar et al. 2024). Achieving this will depend on effectively implementing existing ESG frameworks and developing new ones that prevent socio-environmental conflicts. Centering Indigenous leadership in finance and governance is essential, not only for Indigenous communities, but as a model for more sustainable and equitable coastal life.

## Supplementary Information

Below is the link to the electronic supplementary material.

Supplementary file1 (PDF 421 KB)

## Data Availability

The dataset is available upon request.
