# Supplementary material for: Corporations and financial institutions driving coastal conflicts involving Indigenous Peoples
Source: Ambio. 2026 May 13;55(10):2422–37. doi: 10.1007/s13280-026-02406-x (PMC13554030; doi:10.1007/s13280-026-02406-x)
Supplement: Supplementary file 1 [file 13280_2026_2406_MOESM1_ESM.pdf]

Supplementary material for

Corporations and financial institutions driving coastal conflicts involving Indigenous Peoples

Villasante, S.*, Verba, J. T., Relano, V., Gómez, S., Bakit, J., Bennett, N.J., Charles, A., Calderón-Contreras, R., Cisneros-Montemayor, A.M., Flores, B. M., Garibaldi, L.A., Pennino, M. G., Lopes, P.F.M., Pascual, U., Roskruge, N., Flores-Díaz, A.C., Hernández Márquez, Y., Karen O'Brien

***Corresponding author**

Sebastian Villasante
EqualSea Lab-CRETUS
University of Santiago de Compostela
15782, A Coruña, Spain
E-mail: sebastian.villasante@usc.es

Materials and methods

1. Socio-environmental conflict data collection

To examine socio-environmental conflicts involving Indigenous Peoples in coastal areas, we conducted a targeted case selection from the Environmental Justice Atlas (EJAtlas), a global and collaborative repository of socio-environmental conflict data (Temper et al. 2015; Temper et al. 2018). The EJAtlas is an interactive database that records socio-environmental conflicts worldwide, and its coverage and diversity of cases makes it a suitable way of measuring environmental injustice on a global scale. Coordinated by an international team of researchers, it compiles standardized case information contributed by a global network of collaborators, providing a systematic basis for comparative analysis across regions and sectors. In the EJAtlas, the unit of analysis corresponds to a thoroughly documented case, either in the form of a project-driven campaign or a locally rooted socio-environmental struggle (Temper et al. 2018). These cases are linked to grievances over perceived negative social or ecological impacts, which at times escalate into nationally significant protest events or feed into broader mobilization efforts. In the EJAtlas a conflict is defined as a situation where communities mobilize or express grievances in response to perceived social and/or ecological impacts caused by an economic activity, project, or policy. A case is included only when there is clear social contestation, involving (i) an economic activity, (ii) perceived harm, (iii) affected Indigenous Peoples, and (iv) forms of mobilization such as protests or legal actions. Contestation is expressed through a diverse repertoire of collective action, including media reports, legal proceedings, advocacy campaigns, petitions, assemblies, street demonstrations, boycotts, strikes, threats, civil disobedience, episodes of collective violence, and other related strategies. The inclusion of cases in the atlas follows specific criteria of relevance: recognition by environmental activists and

organizations at the national, regional, or thematic level, visibility and coverage in the media, and the scale of the project, measured in terms of investment, magnitude of impacts, and conflict intensity.

The aim is to represent the most significant cases by country and conflict category. As such, the EJAtlas constitutes a large, purposive dataset, built from expert inputs, of recent and visible ecological distribution conflicts reported globally, though with some unevenness in geographical coverage. Data sources for the documented cases are diverse, ranging from public agencies and local activist groups to academic studies, company websites, as well as blogs, magazines, and newspapers. All sources are systematically cited within the database entries, which remain publicly accessible, ensuring transparency and traceability of the information compiled (Temper et al. 2018).

From the full database (N=4296 in March 2025), we identified documented conflicts located in countries with marine or oceanic coastlines. We then applied a spatial filter, retaining only conflicts within a 30-kilometer buffer from the coastline. This ensured the inclusion of cases directly or indirectly shaped by coastal ecosystems and their associated social-ecological conditions. We focused on conflicts occurring within, overlapping with, or adjacent to territories inhabited or managed by Indigenous Peoples or related communities. This identification relied on multiple complementary sources. The LandMark platform, a global geospatial database of Indigenous and community lands, was used to assess customary tenure within conflict areas. This geospatial analysis was further cross-validated with peer-reviewed literature and ethnographic studies, including Garnett et al. (2018) and Cisneros-Montemayor et al. (2016), which provide comprehensive mappings of Indigenous-governed territories. In cases where geospatial data were unavailable, we relied on the qualitative descriptions in EJAtlas, which frequently include references to Indigenous Peoples, territorial claims, and self-identification as Indigenous or traditional communities. These descriptions were systematically triangulated with supplementary evidence from academic literature, NGO reports, and official government documents. This approach yielded a refined dataset of 401 socio-environmental conflicts intersecting both spatially and socially with Indigenous Peoples' territories, enabling a more precise characterization of their scope, distribution, and impacts in coastal contexts.

The methodology encompassed both direct spatial overlaps between conflict sites and Indigenous lands, and proximate cases with potential to undermine Indigenous rights, governance systems, and relationships with coastal ecosystems. This systematic approach establishes a transparent and replicable framework for case inclusion, providing a robust foundation for subsequent analyses of conflict dynamics, socio-environmental impacts, and community responses. For each socio-environmental conflict, we implemented a systematic identification and classification protocol based on EJAtlas coding, enabling both quantitative and qualitative analyses. Core variables, such as conflict name, country, and UN region (using the second level of categorization: Northern America, Latin America and the Caribbean, Northern Europe, Western Europe, Eastern Europe, Southern Europe, Northern Africa, Sub-Saharan Africa, Western Asia, Southern Asia, Eastern Asia, South Eastern Asia, Australia and New Zealand, Micronesia, Melanesia, Polynesia), were recorded to facilitate spatial visualization and to detect geographic patterns.

Each conflict was categorized according to a first-tier typology widely used in environmental justice research, ensuring comparability across cases. The EJAtlas coding framework was selected for its global coverage, standardized methodology, and extensive validation within the field, which together enhance the reliability and replicability of our classification process. The categories included *Biodiversity conservation*; *Biomass and land use* (e.g., forestry, agriculture, fisheries); *Fossil fuels and climate justice/energy* (which we named as *Energy production*); *Industrial and utility*

development; *Infrastructure and urban expansion*; *Mineral extraction*; *Nuclear issues*; *Tourism and recreation*; *Waste management*; and *Water management*.

Conflict intensity was assessed using the EJAtlas standardized scale (*Latent, Low, Medium, High*, or *Unknown*), informed by indicators such as protest occurrence and magnitude, documented violence, legal proceedings, and involvement of national or international stakeholders. It is important to emphasize that conflict intensity as coded here does not necessarily reflect the level of harm or injustice of the cases in the dataset, and only the extension of the impact. It is possible that it actually reflects the opposite: higher intensity, meaning larger magnitude, might be reflected in more attention from the media or general society, while lower level conflicts can represent cases involving less visible and more repressed parts of the society.

In addition, we extracted from EJAtlas the name of the corporation operating the project connected to the conflicts, along with the locations of their headquarters, to investigate the dynamics between their origins and operations. Corporations could be represented by multinational, national or state institutions of different sizes or sectors. The corporations were coded as operating domestically or abroad. The financial institutions names were also extracted when available, which were represented by international and national institutions such as private banks, state banks, multilateral development banks, among others. Associations between conflict intensity and the involvement of foreign vs domestic corporations were statistically tested using a Chi-squared test in R v4.0107. The effect of the number of corporations operating each project on conflict intensity was tested using the cor.test function in R v4.0, for i) the general dataset, ii) each UN region with four or more occurrences, and iii) each sector.

2. Data limitations

While the EJAtlas provides an invaluable repository for mapping socio-environmental conflicts, it has inherent limitations linked to its design and scope. The database is not an exhaustive inventory of global conflicts but rather a purposive sample, shaped by the visibility of cases in the media, recognition by activist groups, or the scale of investment and impacts. This approach means that smaller-scale conflicts, less publicized struggles, or cases in regions with limited reporting capacity may be underrepresented. As a result, the dataset tends to emphasize highly visible or emblematic conflicts, which may bias comparative analysis toward areas with stronger activist networks or higher media coverage.

Additionally, the uneven geographical coverage of the EJAtlas raises challenges for generalizing findings across regions. Since documentation relies on contributions from a global network of collaborators, data quality and completeness may vary significantly depending on local resources, expertise, and access to information. The reliance on publicly available sources, ranging from NGOs and press coverage to company websites, further introduces variability in reliability and depth of documentation. While transparency is ensured by citing sources, the dependence on secondary information also limits the ability to capture the full complexity of conflicts, particularly informal or less visible dynamics of resistance. Together, these constraints underscore the need to interpret EJAtlas findings as indicative rather than representative of the global landscape of socio-environmental conflicts.

The collaborative nature of the EJAtlas can also include potential biases in the datasets. EJAtlas incorporates information from a diverse range of sources, often provided by civil society organizations voluntarily reporting cases. Although the moderators of the EJAtlas review all

submitted cases, it is difficult to verify all of the information provided. Because of this, it is possible that our dataset presents biases in terms of geographical locations, intensity of the conflicts, and potential mistakes included during the collaborative process. Additional biases may arise from underreporting in politically sensitive contexts, uneven geographic coverage of data sources, and restricted access to information in regions where conflicts are poorly monitored or deliberately obscured. For example, the subjectivity inherent in the EJAtlas conflict intensity scale, which is based on qualitative descriptions of events, may vary across contributors and regions. Although we relied on the standardized EJAtlas categories (*Latent, Low, Medium, High*), differences in reporting detail or interpretation could influence how intensity levels are assigned. Similarly, the classification of projects into economic sectors may be affected by inconsistencies in source documentation or coding practices. While we adhered strictly to the EJAtlas typology to minimize researcher bias, variations in how cases are described or categorized across the database may introduce classification uncertainty.

The EJAtlas, while comprehensive, also has limitations in tracing financial flows, particularly regarding the role of offshore finance and tax haven–based entities that significantly influence extractive projects in Indigenous coastal areas. Confidentiality clauses and non-disclosure agreements further restrict visibility of Indigenous resistance movements and community opposition. Additionally, groups in some regions are threatened and fear making some conflicts visible, limiting the knowledge of socio-environmental conflicts affecting Indigenous Peoples. This fear of reprisals is a key factor that constrains the visibility and reporting of such conflicts.

Besides, the integration of heterogeneous datasets inevitably poses challenges, and the findings of this study should therefore be interpreted with caution. The number of Indigenous Peoples identified as affected by economic projects represents a conservative estimate, constrained by gaps in the availability, accuracy, and completeness of information regarding both the formal recognition of Indigenous lands and the documentation of Indigenous presence. As such, our figures likely underrepresent the true scale of Indigenous communities currently impacted worldwide, given the lack of comprehensive and systematic data.

The geographic attribution of ethnic groups in this study relied on cross-referencing of multiple authoritative sources (e.g., LandMark and IWGIA), which may introduce spatial inaccuracies due to the limited precision of available location data. Nevertheless, the resulting mapped distribution of Indigenous populations, when combined with records of documented conflicts, offers a valuable and systematic approximation of spatial patterns of impacts. Still, important data gaps persist in certain regions such as Mexico, where chronic underreporting obscures both the presence and scale of conflicts affecting Indigenous groups. Finally, our use of a 30 km buffer to define coastal conflicts introduces a methodological limitation. While this criterion provides a systematic and replicable approach to case selection, it may exclude Indigenous communities located beyond this distance but still dependent on marine ecosystems, and conversely, may include conflicts not directly linked to coastal processes. As such, the buffer should be regarded as a pragmatic but imperfect proxy for identifying coastal socio-environmental conflicts. Taken together, these limitations suggest that our results represent a conservative baseline. The patterns identified here are therefore likely to underestimate, rather than overstate, the extent and intensity of coastal socio-environmental conflicts affecting Indigenous communities.

References

Cisneros-Montemayor, A. M., D. Pauly, L. V. Weatherdon, and Y. Ota. 2016. A Global Estimate of Seafood Consumption by Coastal Indigenous Peoples. *PLOS ONE* 11. Public Library of Science: e0166681. doi:10.1371/journal.pone.0166681.

Garnett, S. T., N. D. Burgess, J. E. Fa, Á. Fernández-Llamazares, Z. Molnár, C. J. Robinson, J. E. M. Watson, K. K. Zander, et al. 2018. A spatial overview of the global importance of Indigenous lands for conservation. *Nature Sustainability* 1. Nature Publishing Group: 369–374. doi:10.1038/s41893-018-0100-6.

Temper, L., D. del Bene, and J. Martinez-Alier. 2015. Mapping the frontiers and front lines of global environmental justice: the EJAtlas. *Journal of Political Ecology* 22. University of Arizona Libraries. doi:10.2458/v22i1.21108.

Temper, L., F. Demaria, A. Scheidel, D. Del Bene, and J. Martinez-Alier. 2018. The Global Environmental Justice Atlas (EJAtlas): ecological distribution conflicts as forces for sustainability. *Sustainability Science* 13: 573–584. doi:10.1007/s11625-018-0563-4.
